# Methaemoglobinaemia: From pathophysiology to contemporary clinical management

**DOI:** 10.1111/bjh.70647

**Published:** 2026-07-01

**Authors:** Alexander J. Twine, David C. Rees

**Affiliations:** ^1^ Faculty of Life Sciences & Medicine King's College London London UK; ^2^ School of Cancer and Pharmaceutical Sciences, Faculty of Life Sciences and Medicine King's College Hospital London UK

**Keywords:** cyanosis, inherited enzymopathy, methaemoglobinaemia

## Abstract

Methaemoglobin (MetHb) is an oxidised form of haemoglobin (Hb) unable to bind oxygen. Raised levels of MetHb reduce the blood's oxygen‐carrying capacity, causing potentially severe hypoxaemia and possible death. The condition arises from three main pathologies: mutations in globin genes causing Haemoglobin‐M, inherited deficiency of the enzyme cytochrome b5 reductase (CytB5R) responsible for reducing MetHb back into functional Hb, and exposure to some oxidising agents. Well‐documented causative agents include dapsone and cocaine‐derived anaesthetics, with emerging evidence highlighting an increasing contribution from recreational and non‐prescribed exposures. As the concentration of MetHb level increases, the oxygen‐carrying capacity of the blood decreases. MetHb levels are usually measured by co‐oximetry. Patients are typically asymptomatic at MetHb concentrations <10% with symptoms developing as levels increase, including cyanosis, confusion, arrhythmias, coma and death. These features will present alongside misleadingly normal partial pressure of oxygen in arterial blood and arterial oxygen saturation values on arterial blood gas and will not improve with supplemental oxygen. Management depends on severity, with intravenous methylene blue remaining first‐line treatment for symptomatic cases. Alternative therapies include high‐dose vitamin C, exchange transfusion and potentially hyperbaric oxygen, although there is little evidence to suggest how these should be used. Due to the potentially confusing acute presentation of the condition, the diagnosis can easily be missed.

**Abbreviations**
ABG: arterial blood gas
CytB5: cytochrome b5
CytB5R: cytochrome b5 reductase
Cyt P450: cytochrome P450
DDS‐NOH: dapsone hydroxylamine
G‐6‐P: glucose‐6‐phosphate
G6PD: glucose‐6‐phosphate dehydrogenase
Hb: haemoglobin
HbA: adult haemoglobin (α_2_β_2_)
HbF: fetal haemoglobin (α_2_γ_2_)
HbM: Haemoglobin M
MADDS‐NOH: monoacetyldapsone hydroxylamine
MetHb: methaemoglobin
NAD: nicotinamide adenine dinucleotide (oxidised form)
NADH: nicotinamide adenine dinucleotide (reduced form)
NADP: nicotinamide adenine dinucleotide phosphate (oxidised form)
NADPH: nicotinamide adenine dinucleotide phosphate (reduced form)
PaO_2_
: partial pressure of oxygen in arterial blood
RCM: recessive congenital methaemoglobinemia
Ribose‐5‐P: RIBOSE‐5‐phosphate
SaO_2_
: arterial oxygen saturation
SpO_2_
: peripheral oxygen saturation

## INTRODUCTION

Methaemoglobinaemia is a disorder of the blood where haemoglobin (Hb) is oxidised to methaemoglobin (MetHb).^1^ MetHb contains ferric iron (Fe^3+^) rather than the ferrous iron (Fe^2+^) required for reversibly binding oxygen.^1^ MetHb can therefore not carry oxygen and impairs oxygen delivery to tissues. Patients suffer progressively more severe functional anaemia as MetHb concentrations increase; this can be life threatening if not promptly recognised and managed.^2^ This disorder is often missed in clinical practice and can be chronic, with steady state high levels as a result of enzyme deficiencies or Hb mutations, or it can be acquired with acute increase in levels triggered by exposure to oxidising agents.^3^, ^4^, ^5^ Recent epidemiological data suggest that the pattern of causative exposures may be evolving, with an increasing contribution from non‐prescribed and recreational agents in contemporary clinical practice.

This review explores established physiological and biochemical concepts, with recent clinical evidence to provide a contemporary overview of methaemoglobinaemia. The pathophysiology, presentation, diagnostic approach and current management recommendations are discussed alongside areas of ongoing uncertainty and priorities for future research.

## METHODOLOGY

This narrative review summarises the published literature relating to the pathophysiology, investigation and management of methaemoglobinaemia. Relevant English‐language publications were identified through searches of PubMed and Google Scholar up to April 2026. Search terms included “methaemoglobinaemia”, “methaemoglobinemia”, “metHb” and related terms. Additional relevant publications were identified through citation mining of key articles. The literature was reviewed and presented as a narrative synthesis.

## PATHOPHYSIOLOGY

### Physiological methaemoglobin production

Normal haemoglobin (HbFe^2+^) contains a haem pocket: a hydrophobic group with two key histidine residues, a protoporphyrin ring and a central Fe^2+^.^6^ The haem pocket facilitates the reversible binding of oxygen and protects the Fe^2+^ from oxidation.^7^ Despite these adaptations, auto‐oxidation of Fe^2+^ in oxyhaemoglobin to Fe^3+^ occurs at a rate of about 3% /day.^8^, ^9^ The offending reaction is shown below^10^:

$${\text{HbFe}}^{2+}O_{2}\to {\text{HbFe}}^{3+}+O_{2}•$$

**FIGURE 1:**
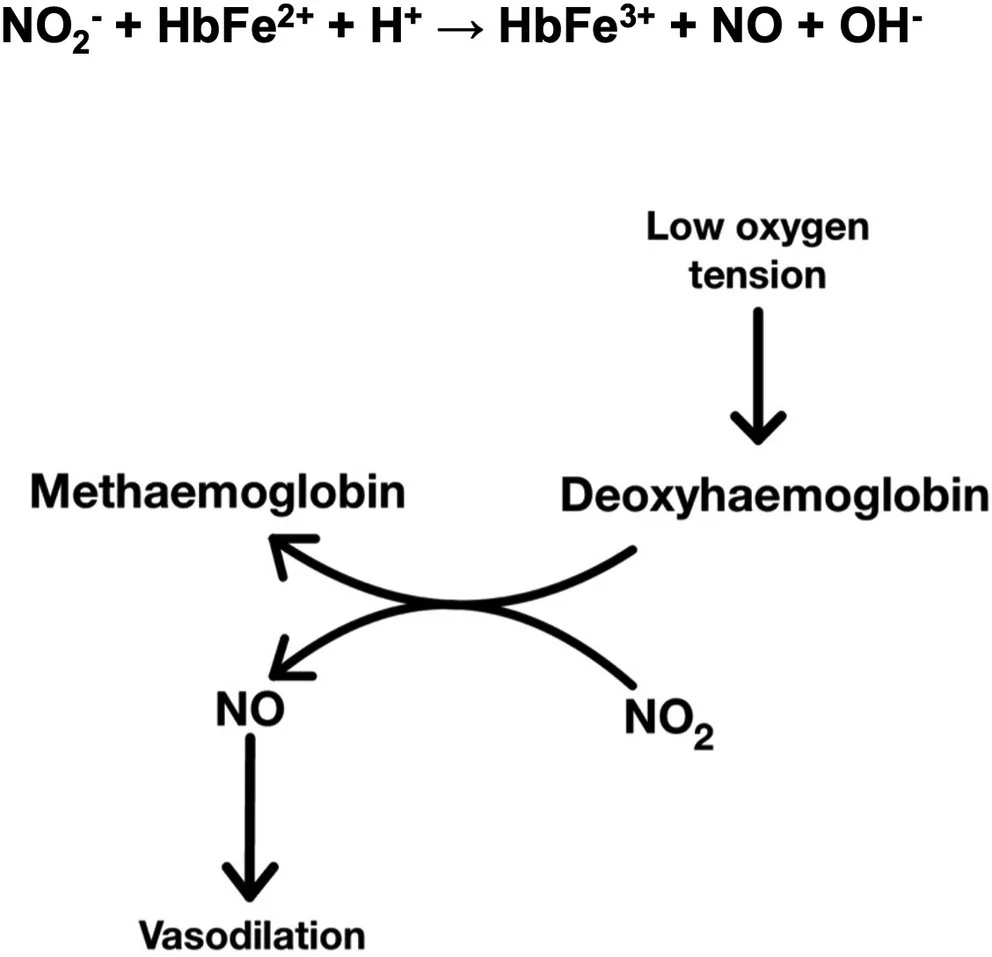
Deoxyhaemoglobin reduces NO_2_ into NO, leading to MetHb production and vasodilation.^13^ NO, nitric oxide; NO_2_, nitrite.

Some adaptive features of MetHb have been suggested, including a role in mediating vasodilation in areas of ischaemia.^11^ Nitrite (NO_2_) readily oxidises deoxyhaemoglobin (HbFe^2+^) to MetHb (HbFe^3+^) producing nitric oxide (NO): a key mediator of vasodilation (Figure 1).^12^, ^13^

This reaction does not occur with oxyhaemoglobin as iron is stabilised in the Fe^2+^ state and so lacks the required reducing power.^11^, ^14^ As concentrations of deoxyhaemoglobin are higher in areas of low oxygen tension, the reduction of NO_2_ by HbFe^2+^ acts as an oxygen sensor, eliciting vasodilation and increased blood flow to these areas.^13^

### Physiological methaemoglobin reduction

When Fe^2+^ is oxidised to Fe^3+^ in MetHb, it does not have an electron available to share and reversibly bind with oxygen.^15^ It is therefore essential that the body has effective mechanisms to reduce MetHb.

Physiological reduction mechanisms keep the concentration of MetHb below 1%.^15^

Cytochrome b5 reductase (CytB5R) is responsible for the majority of MetHb reduction.^16^ Two forms of this enzyme are present in humans: an erythrocyte‐restricted form and a global, membrane‐bound form.^17^ Other minor pathways play a role in reducing MetHb, including nicotinamide adenine dinucleotide phosphate (reduced form) (NADPH)‐diaphorase: this system usually accounts for <5% of MetHb reduction but is massively potentiated by methylene blue.^18^

The above physiology reveals three opportunities for pathology leading to methaemoglobinaemia:

1. Inherited CytB5R deficiency.^4^
2. Mutations in the globin genes which destabilise the haem molecule leaving it vulnerable to oxidation: Haemoglobin‐M disease.^5^
3. Acquired methaemoglobinaemia by exposure to oxidising agents.^3^

### Recessive congenital methaemoglobinaemia

While investigating clinical uses for vitamin C in 1943, Dr. James Deeny discovered two blue‐skinned brothers in Northern Ireland: Fred and Russell Martin.^19^ Treatment with high‐dose vitamin C rendered Fred's cyanosis barely detectable and, after excluding cardiac causes, researchers diagnosed ‘familial idiopathic methaemoglobinaemia’, now known as recessive congenital methaemoglobinaemia (RCM).^19^ Henry Barcroft then discovered a mismatch between the Hb level and oxygen‐carrying capacity of Russel's blood, due to raised levels of MetHb.^20^

**FIGURE 2:**
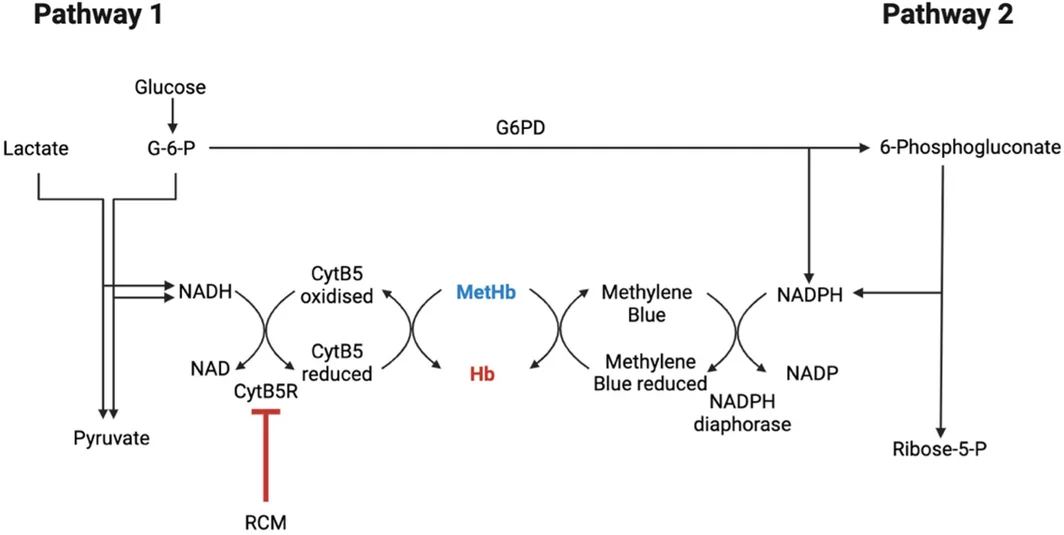
Summary of methaemoglobin reduction pathways described by Gibson.^15^, ^21^ Pathway 1 uses glycolysis or lactate dehydrogenase to produce NADH as a substrate for CytB5R to reduce CytB5. Reduced CytB5 then reduces MetHb into functional Hb. Pathway 2 uses NADPH from the pentose phosphate pathway to reduce administered methylene blue. Reduced methylene blue then reduces MetHb. In RCM, patients are deficient in CytB5R and are ineffective at reducing MetHb. CytB5, cytochrome b5; CytB5R, cytochrome b5 reductase; Hb, haemoglobin; MetHb, methaemoglobin; NADH, nicotinamide adenine dinucleotide; NADPH, nicotinamide adenine dinucleotide phosphate; RCM, recessive congenital methaemoglobinaemia.

Quentin Gibson later investigated the pathophysiology of this disease by studying the reduction of MetHb using methylene blue in the blood of a healthy individual, and a patient with RCM.^21^ He suggested two pathways for the reduction of MetHb (Figure 2). In pathway 1, glucose or lactate can be used as the substrate to reduce nicotinamide adenine dinucleotide (NAD) to nicotinamide adenine dinucleotide (reduced form) (NADH), whereas pathway 2 (pentose phosphate pathway) uses glucose to reduce nicotinamide adenine dinucleotide phosphate (oxidised form) (NADP) to NADPH. NADH and NADPH can then indirectly reduce MetHb. Gibson found that in the presence of methylene blue both the patient's blood and the healthy blood could use glucose as a substrate to reduce MetHb.^21^ However, unlike the healthy blood, the patient's blood could not utilise lactate.^21^ Therefore, it was suggested that the patient's blood relied on pathway 2 due to a defect in pathway 1. Gibson suggested that a deficiency in ‘coenzyme factor 1’, now known as CytB5R, was responsible.^21^ The patient's blood was ineffective at reducing MetHb without the addition methylene blue to potentiate pathway 2.^21^

The mutations responsible for CytB5R deficiency leading to RCM in the original cases described by Deeny were identified in 2002 by Percy et al.^4^ *CYB5R3* is the gene coding for both forms of CytB5R, with the first exon of *CYB5R3* encoding a membrane binding domain.^17^ Two transcripts are generated from *CYB5R3*: M and S.^17^ The S transcript does not contain the first exon, producing a water‐soluble version of the enzyme, devoid of the membrane binding domain, only found in erythrocytes.^17^ The M transcript—complete with the first exon—produces a membrane‐bound form of the enzyme found in the endoplasmic reticulum and the inner mitochondrial membrane.^17^ RCM—the result of mutations in *CYB5R3*—is classified into two main types:

1. Type 1 is a deficiency in water‐soluble CytB5R only.^22^
2. Type 2 is a global deficiency in CytB5R.^2^, ^22^

Type 1 is typically the result of missense mutations in *CYB5R3* leading to structural instability of the enzyme.^15^ Intracellular MetHb concentrations increase throughout the affected erythrocyte's life due to their inability to regenerate protein.^23^ Patients with type 1 CytB5R deficiency appear visibly cyanosed due to impaired oxygen delivery to the skin and mucous membranes.^22^

Type 2 is typically the result of deletions or truncated transcripts due to premature stop codons.^15^, ^22^ These mutations cause global loss of CytB5R, affecting lipid metabolism in multiple tissues, critically in the central nervous system (CNS) with patients suffering severe progressive neurological and cognitive impairment.^2^, ^24^, ^25^, ^26^

Although heterozygous CytB5R carriers are usually clinically well, recent guidance advises the avoidance of precipitating oxidant exposures in these individuals. It has been recommended to test the first‐degree relatives of patients with homozygous CytB5R deficiency to identify carriers.^27^

### Haemoglobin M disease

Disturbance to the delicate configuration of globin proteins discussed above can leave Fe^2+^ vulnerable to oxidation.^5^ These disturbed proteins—termed ‘Haemoglobin M’ (HbM)—result from amino acid substitutions affecting the structure of the hydrophobic haem pocket, most frequently one of the key histidine residues.^5^, ^28^, ^29^, ^30^, ^31^, ^32^ Two out of nine reported mutations resulted in substitution of other amino acids.^28^, ^33^ The result of these mutations is stabilisation of Fe^3+^.^33^ These structural changes also prevent CytB5R from effectively reducing MetHb, producing persistent methaemoglobinaemia.^34^

All reported cases of HbM disease are heterozygous dominant, meaning less than 50% of these patients' total Hb is HbM.^33^ These patients have functional anaemia and can appear cyanosed; however, patients adapt to the level of MetHb and there are enough oxygen‐carrying globin chains to support life.^29^ Homozygous HbM has not been described and is probably not compatible with life, causing in utero death (α‐ and γ‐globin variants) or death in the first year of life (β‐globin variants). Patients with HbM disease have mutations in either the (α‐, β)‐ or γ‐globin chains. α‐globin variants include HbM Boston and HbM Iwate; β‐globin variants include HbM Saskatoon and HbM Hyde Park; and γ‐globin variants include HbFM Osaka and HbFM Fort Ripley.^28^, ^29^

Fetal haemoglobin (HbF, ααγγ) is replaced by adult haemoglobin (HbA, ααββ) shortly after birth, and the pattern of disease reflects this^35^:

- Mutations in the α‐globin are pathological from birth and throughout life.^5^, ^32^
- γ‐globin mutations affect foetuses and newborns, but MetHb levels decrease rapidly over the first 6 months of life as HbF is replaced by adult HbA.^28^, ^29^
- β‐globin mutations lead to cyanosis around 3 months after birth.^30^, ^31^

Recent laboratory studies have shown that HbM variants can interfere with co‐oximetry by producing abnormal light absorption patterns: some α‐globin variants can mimic sulfhaemoglobin, while others (e.g. HbM Saskatoon) can result in unreliable or uninterpretable MetHb measurements. HbM variants may be electrophorectically silent and diagnosis usually involves Deoxyribonucleic acid (DNA) analysis of the globin genes. Patients with these variants do not require treatment for cyanosis if they are otherwise well as they are clinically compensated. Recognition of these patterns can prevent unnecessary anti‐dotal therapy, and prompt appropriate molecular diagnostic confirmation and family counselling.^36^

### Exposure to oxidising agents

**FIGURE 3:**
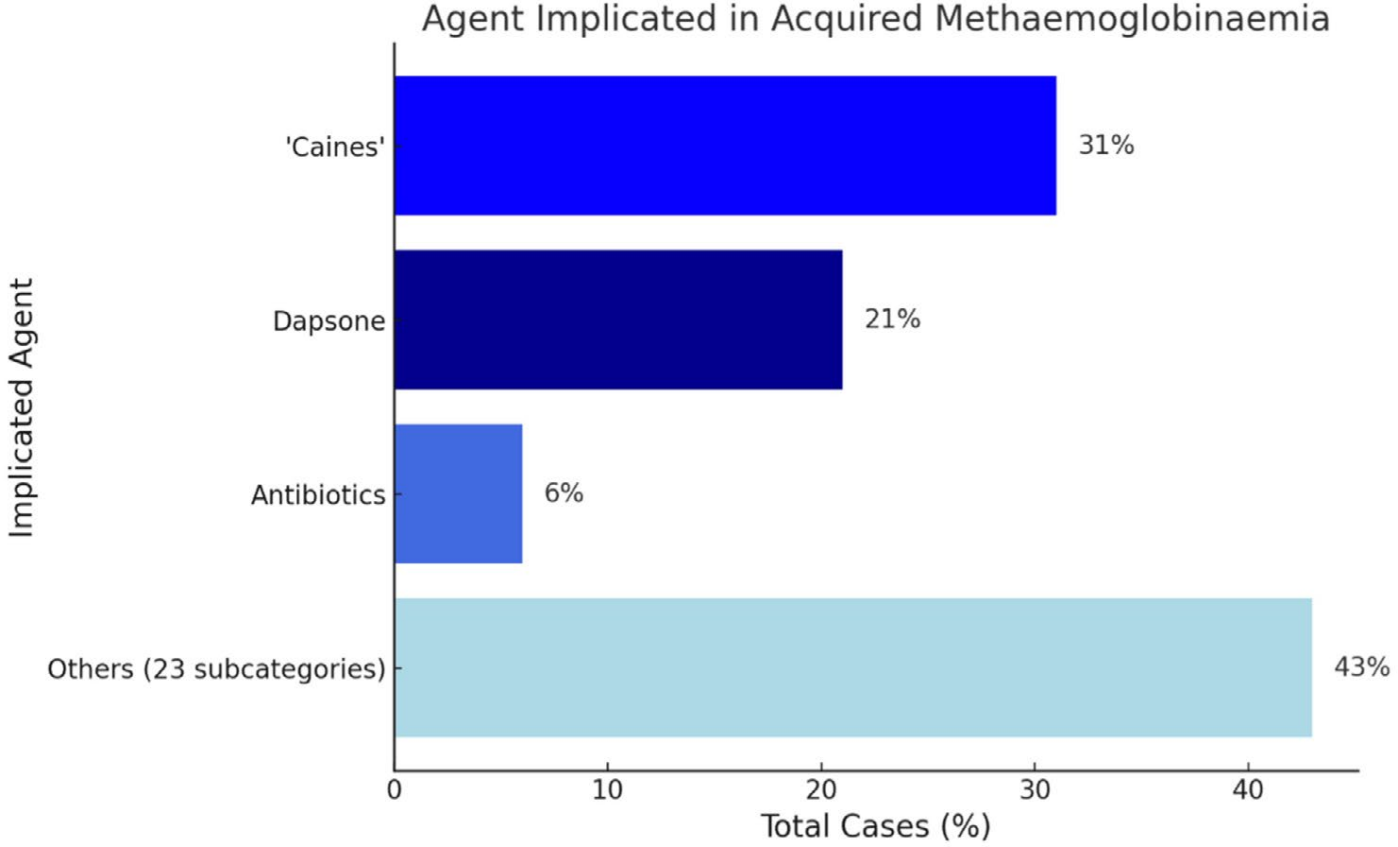
Summary of findings by Gao et al: causes of reported cases of acquired methaemoglobinaemia. Benzocaine‐derived anaesthetics (caines) and dapsone make up over half the reported cases.^37^

Acute methaemoglobinaemia can be acquired following exposure to oxidising agents. A systematic review of reported cases between 1980 and 2020 identified local anaesthetics (‘‐caine’ agents) and dapsone as the most frequently implicated causes (Figure 3).^37^ More recent data from a large urban toxicology centre suggest a shift in this pattern, with volatile nitrites emerging as the most commonly reported cause.^38^ This likely reflects an increasing contribution from recreational and community exposures, although such findings may be influenced by regional patterns of drug use and referral bias.

Methaemoglobinaemia caused by local anaesthetics most commonly occurs following mucosal administration, such as during intubation or trans‐oesophageal echocardiography.^37^ These agents, along with dapsone, contain an aniline group and are thought to be metabolised to hydroxylamines, which are potent oxidisers of Hb.^11^, ^39^

**FIGURE 4:**
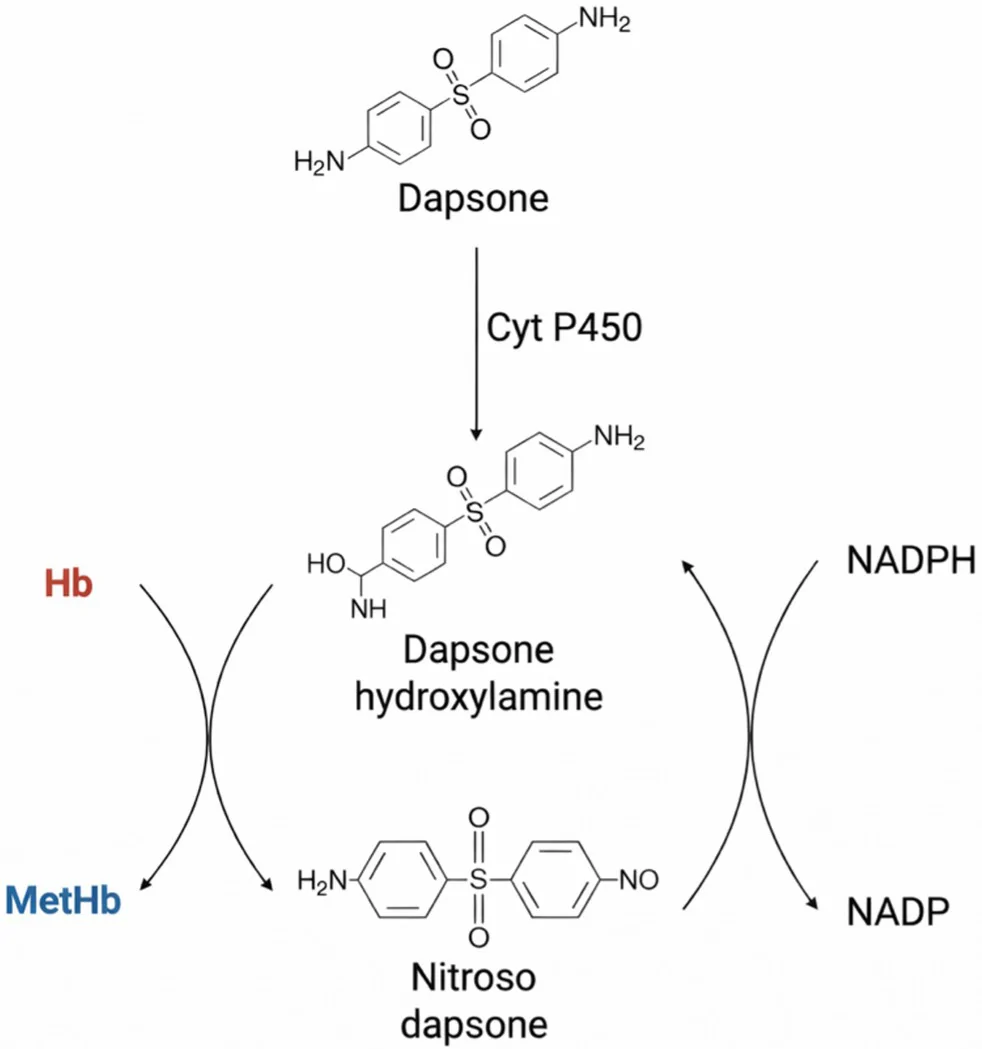
Recycling of dapsone metabolites.^41^, ^42^, ^43^ Hydroxylamines oxidise haemoglobin into methaemoglobin and are reduced to nitroso‐compounds. Hydroxylamines are regenerated with NADPH. NADPH, nicotinamide adenine dinucleotide phosphate.

Dapsone remains a well‐characterised and clinically significant cause of methaemoglobinaemia. A 2024 retrospective cohort study found dapsone responsible for the majority of clinically significant acquired MetHb cases.^3^ Dapsone is metabolised by cytochrome P450 into two hydroxylamine metabolites: dapsone hydroxylamine (DDS‐NOH) and monoacetyldapsone hydroxylamine (MADDS‐NOH).^40^ Scott and Rasbridge found significant MetHb formation when erythrocytes were incubated with either of these two metabolites, but not with unmetabolised dapsone.^41^ The suggested mechanism of MetHb formation with hydroxylamines is a coupled oxidation reaction (Figure 4).^41^, ^42^, ^43^ They found that more MetHb was formed than expected, possibly through direct Hb oxidation by each hydroxylamine molecule.^41^ It was also found that less MetHb was formed in the absence of glucose and in glucose‐6‐phosphate dehydrogenase (G6PD)‐deficient blood.^41^ These findings support an earlier suggestion by Kiese that the nitroso‐compounds formed in this reaction are recycled back to hydroxylamines through direct reduction by NADPH (Figure 4) from the pentose phosphate pathway (requiring glucose and G6PD, Figure 2).^41^, ^44^

The cycle suggested by Kiese gives each molecule of hydroxylamine metabolite a great oxidation potential against Hb.^44^ It has been shown in‐vitro that benzocaine‐hydroxylamine oxidises Hb in a similar fashion to DDS‐NOH.^39^ Studies on the metabolism of benzocaine to produce benzocaine‐hydroxylamine are ongoing.^39^

Recent clinical epidemiological studies have highlighted the growing importance of non‐prescribed and recreational exposures in acquired methaemoglobinaemia. Severe cases of MetHb resulting in death or life‐threatening complications were associated with recreational alkyl nitrites and, increasingly, with suicide attempts involving sodium nitrite.^3^ Sodium nitrite is a widely available meat preservative—readily accessible for purchase online. Ingestion of as little as 1 gram can precipitate life‐threatening methaemoglobinaemia.^45^ Recent data suggest a marked rise in sodium nitrite‐related deaths over the past decade. A 2024 US study reported no recorded deaths between 2000 and 2018, followed by a steady yearly increase from 2019 to 2022.^46^ This trend has been attributed, in part, to increased online availability and dissemination of information regarding its use for self‐harm.^46^ Other recreational drugs have been linked to acquired methaemoglobinaemia, including nitrous oxide (laughing gas) and cocaine when adulterated with oxidising agents such as phenacetin and local anaesthetics. Volatile nitrites and adulterated cocaine have been documented to cause very high MetHb levels of up to 90%, with some fatalities.^3^, ^47^

Multiple recent systematic reviews have associated rasburicase—used for the treatment of tumour lysis syndrome—with the development of methaemoglobinaemia, particularly in patients with G6PD deficiency.^48^, ^49^ This is thought to result from the generation of hydrogen peroxide during uric acid metabolism, leading to oxidative stress within erythrocytes. A 2024 review emphasised the importance of screening for G6PD deficiency prior to rasburicase administration. Where this is not feasible, such as in acute cases, close monitoring for evidence of MetHb is recommended during treatment.^48^ As discussed below, methylene blue should be avoided in patients with G6PD deficiency due to the risk of precipitating haemolysis: ascorbic acid may be a beneficial alternative in these cases.^48^

Other drugs implicated in precipitating methaemoglobinaemia include glyceryl trinitrate, nitric oxide, primaquine, silver nitrate, sulphadiazine, co‐trimoxazole and sulphasalazine. Exposure to nitrates from drinking well water has also been described as causing high levels of MetHb. In most cases this occurs in people with low activity of CytB5R, including neonates and those with inherited deficiencies.^1^, ^11^, ^27^

### Functional anaemia

The toxic effects of MetHb are higher than those produced by an equivalent amount of anaemia, suggesting an additional pathological process.^50^ Darling et al. found increasing concentrations of MetHb produce a leftward shift of the oxygen dissociation curve in vitro.^50^ It was suggested that Fe^3+^ increases the oxygen affinity of the remaining Fe^2+^, impairing oxygen release at tissues.^50^ The decreased binding and release of oxygen exacerbate the functional anaemia seen in these cases.

## DIAGNOSIS

### Presentation

**FIGURE 5:**
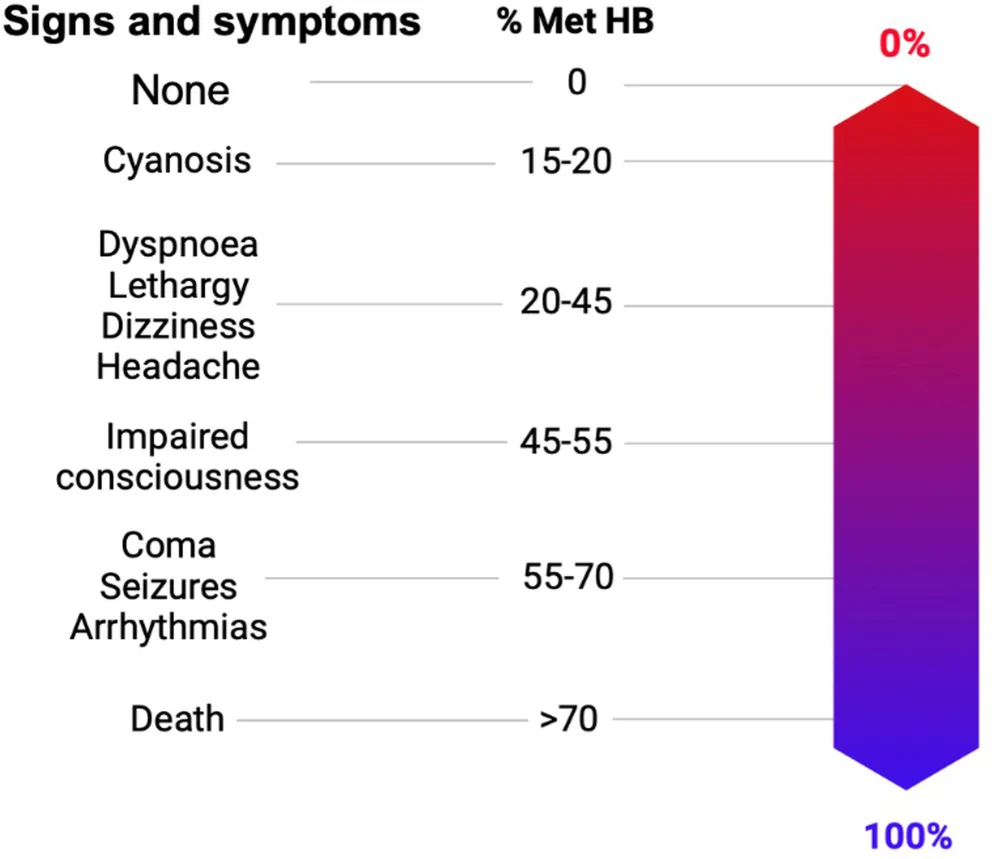
Methaemoglobin concentration and associated signs and symptoms.^1^, ^33^, ^51^ MetHb concentration is represented as a percentage of total Hb; cases may present with more severe signs in patients with anaemia or circulatory dysfunction: this is only a guide. Hb, haemoglobin; MetHb, methaemoglobin.

Patients with methaemoglobinaemia typically present with cyanosis unresponsive to oxygen: this is usually the first sign to develop with increasing concentrations of MetHb.^33^ Figure 5 summarises some signs and symptoms seen as MetHb concentrations increase.^1^, ^33^, ^51^

Patients with congenital methaemoglobinaemia usually present with cyanosis at birth, unresponsive to oxygen therapy.^1^ Oxygen‐unresponsive cyanosis after infancy is therefore likely to have been acquired.

### Investigations

**FIGURE 6:**
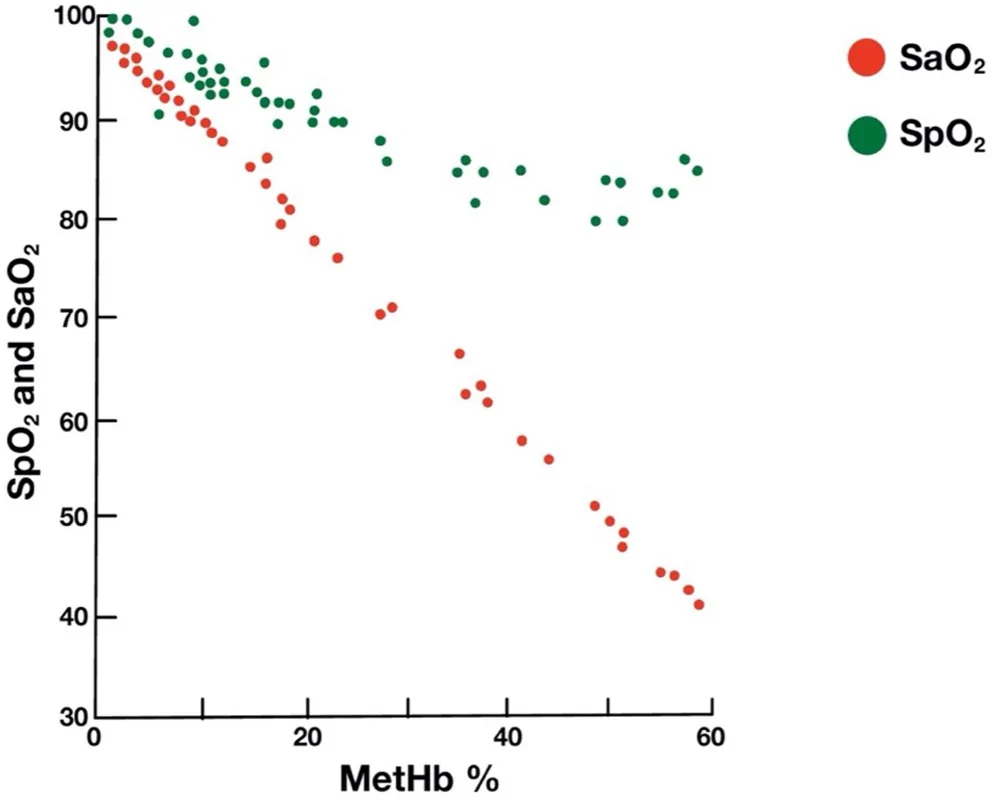
Summary of findings from Barker.^54^ As MetHb concentrations increase, SpO_2_ from pulse oximetry decreases until a plateau at 85% oxygen saturations. SaO_2_ from co‐oximetry decreases linearly as MetHb concentrations increase. MetHb, methaemoglobin; SaO_2_, arterial oxygen saturation; SpO_2_, peripheral oxygen saturation.

Arterial oxygen saturation (SaO_2_) is a measure of oxygen saturation, often calculated from dissolved oxygen partial pressure of oxygen in arterial blood (PaO_2_) on arterial blood gas (ABG).^52^ As methaemoglobinaemia does not affect the ability of oxygen to diffuse into plasma, PaO_2_ remains normal and SaO_2_ inaccurately appears normal or elevated on ABG.^52^ Oxygen saturations are also unreliable as measured by pulse oximetry peripheral oxygen saturation (SpO_2_) in cases of methaemoglobinaemia.^53^ Pulse oximeters use a ratio of two wavelengths of light which are absorbed differently by oxyhaemoglobin and deoxyhaemoglobin: 660:940 nm.^53^ An absorbance ratio of 1:1 indicates 85% oxygen saturation.^53^ A lower ratio indicates higher saturation, and a higher ratio indicates lower saturation. MetHb absorbs these two wavelengths equally, therefore giving a saturation reading of 85%^53^ (Table 1). This supports the work of Barker, who investigated the effect of increasing MetHb concentrations on SpO_2_ reported by pulse oximetry in dogs.^54^ It was found that as MetHb concentrations increased, the reported SpO_2_ decreased until it plateaued at 85% oxygen saturations with a MetHb concentration of >35%^54^ (Figure 6).

**TABLE 1:** Summary of investigations commonly used to assess the oxygen content of the blood in cases of methaemoglobinaemia.

Parameter	What it shows	How it is measured	Value in methaemoglobinaemia	Advantages	Limitations
PaO_2_ (ABG)	Dissolved O_2_ in plasma	Direct measurement by blood gas electrode	Normal to high	Measures pH, PCO_2_ and PaO_2_ directly	Only reflects plasma‐dissolved oxygen
SaO_2_ (ABG)	O_2_ saturation (calculated)	Estimated using PaO_2_ and oxygen dissociation curve	Falsely normal to high	Rapid; routinely available	Unreliable if abnormal Hb present
SpO_2_ (pulse oximetry)	O_2_ saturation	Transcutaneous absorbance at 660 and 940 nm	Fixed at around 85% when MetHb >15%–20%	Non‐invasive; rapid; continuous bedside monitoring	Inaccurate with MetHb; Plateaus around 85%
SaO_2_ (co‐oximetry)	O_2_ saturation	Direct measurement using multiple wavelengths (≥4)	Low	Gold standard; directly measures several different types of Hb	Requires lab equipment

*Note*: Pulse oximetry and ABG are inaccurate investigations in cases of MetHb. Co‐oximetry confirmed with potassium cyanide should be used for diagnosis.^55^

Abbreviations: ABG, arterial blood gas; Hb, haemoglobin; MetHb, methaemoglobin; PaO_2_, partial pressure of oxygen in arterial blood; PCO_2_ partial pressure of carbon dioxide; SaO_2_, arterial oxygen saturation; SpO_2_, peripheral oxygen saturation.

Despite its inaccuracy, the ‘saturation gap’—created by high saturation on ABG (~98%) alongside low saturations clinically and on pulse oximetry (~85%)—has traditionally been regarded as a helpful red flag in identifying possible methaemoglobinaemia.^56^ However, recent evidence suggests it should be considered supportive rather than a universal hallmark. A 2022 systematic review of acquired cases found that a SaO_2_:SpO_2_ ratio >1 was not consistently observed.^37^ Co‐oximetry remains the diagnostic standard in cases where MetHb is clinically suspected.

**FIGURE 7:**
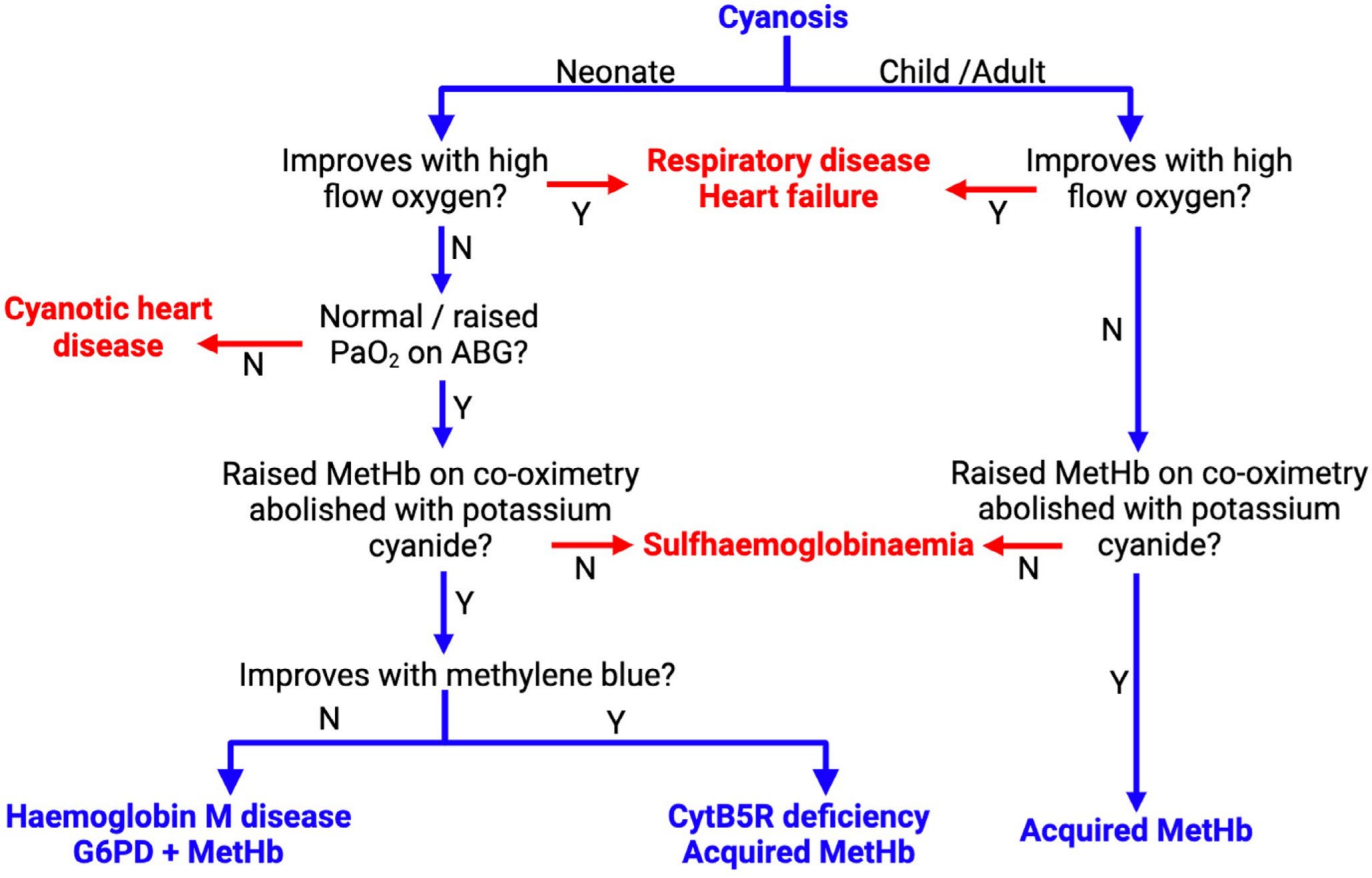
Investigating new cyanosis. Diagnosing methaemoglobinaemia (blue) and differential diagnoses (red).^1^

SaO_2_ reported by co‐oximetry uses 4–8+ different wavelengths of light and provides an accurate measure of oxygen saturation in these cases.^55^ Co‐oximetry can also be used to estimate MetHb concentration in the blood: MetHb has peak absorbance of light at 635 nm.^57^ Sulfhaemoglobin has a similar chocolate‐brown appearance to MetHb and a peak absorbance of 614 nm: this can be misinterpreted as MetHb on older co‐oximeters.^55^, ^57^ As such, co‐oximeter readings should be interpreted with caution until confirmed by the addition of potassium cyanide, which reacts with chocolate‐brown MetHb to produce bright‐red cyanomethaemoglobin. A suggested pathway for the diagnosis of MetHb is given in Figure 7. Evelyn found that the 635 nm absorption band is almost completely abolished by the conversion of MetHb into cyanomethaemoglobin.^57^ Sulfhaemoglobin does not react with potassium cyanide, and so stays chocolate‐brown, with unchanged co‐oximetry.^57^ As discussed, HbM variants should always be considered in clinically compensated individuals who are persistently cyanosed, even when co‐oximetry results are atypical.^36^

Methylene blue, a powerful reducing agent, will resolve cyanosis due to CytB5 reductase deficiency or exposure to oxidising agents by potentiating the NADPH diaphorase pathway (Figure 2).^58^ Methylene blue is ineffective at resolving cyanosis in patients with HbM disease due to structural changes in globin proteins.^59^

## MANAGEMENT

### Congenital methaemoglobinaemia

Patients with CytB5R deficiency typically maintain ‘tolerable’ MetHb concentrations of approximately 15%–30%.^15^ Management is generally conservative unless patients are symptomatic or MetHb levels rise acutely.

Treatment is indicated in symptomatic disease, or acute increases in MetHb levels.

Intravenous methylene blue remains the first‐line therapy for symptomatic patients without G6PD deficiency: typically administered at 1–2 mg/kg as a 1% solution over 5–10 minutes.^60^ High‐dose vitamin C may be used as an adjunct or alternative therapy, particularly in milder cases or where methylene blue is contraindicated, although its onset is slower: taking up to 24 h to lower MetHb levels.^15^, ^60^ In addition to avoiding methylene blue in G6PD deficiency, it should be used with caution in pregnancy and in people with severe renal impairment, a history of severe allergy to thiazide dyes and in those taking some drugs, including fluoxetine and other serotonergic anti‐depressants, tramadol and linezolid.^58^ Vitamin B_2_ has also been described as an adjunctive therapy in chronic management with reported dosing of 20–30 mg/day although supporting evidence remains limited and largely derived from case reports and small series.^60^ Exchange transfusion is not established as a routine strategy for the chronic management of RCM. Its use is generally limited to acute or life‐threatening presentations, particularly when MetHb levels are markedly elevated (e.g. >60%–70%) or when there is an inadequate response or contraindication to methylene blue. In these settings, exchange transfusion may provide rapid reduction in circulating MetHb.^61^

There is no specific treatment for very severe forms of RCM associated with progressive neurological impairment.^15^

At present, there is no effective treatment for Hb‐M disease, other than emergency exchange transfusion if levels have increased acutely or are associated with symptoms.^59^ Bone marrow transplantation could be considered in very severe cases, and stimulation of HbF production could potentially offer benefits for beta‐globin HbMs—further research into possible therapies is warranted.

### Acute methaemoglobinaemia

Treatment of acute methaemoglobinaemia is usually indicated in symptomatic patients with concentrations of MetHb >20%, or in asymptomatic patients >30%.^1^, ^18^ The degree to which symptoms occur depends on the rate at which the level of MetHb increases, with slowly increasing levels being better tolerated.

The causative agent should be identified and stopped or removed. Methylene blue remains first line treatment—following the dose regimen described above (*Congenital methaemoglobinaemia*).^18^ Recent observational data from a large toxicology cohort (*n* = 185) support this approach, demonstrating clinical improvement in 98% of cases, with most patients responding to a single dose within the 1–2 mg/kg range. Repeat dosing was required in approximately 11% of cases, most commonly in association with dapsone or volatile nitrite exposure, likely reflecting ongoing oxidant stress or metabolite recycling. Adverse effects attributable to methylene blue were uncommon (4.9%), with haemolysis reported rarely.^38^ Maintenance dextrose should be provided for all patients receiving methylene blue therapy to supply the pentose phosphate pathway (Figure 2).^1^ If methylene blue fails to reduce MetHb concentrations in the blood, repeated administrations should be used cautiously: >7 mg/kg can cause cyanosis and haemolytic anaemia.^62^ Methylene blue overdose can be disguised as methaemoglobinaemia, potentially leading to further doses of methylene blue being administered: co‐oximetry should be used to assess improvements in oxygen saturations and MetHb concentration to prevent this.^18^, ^62^

G6PD deficiency is a predisposing factor to develop methaemoglobinaemia due to increased oxidative stress; however, methylene blue is contraindicated in these cases.^63^ Not only is it ineffective due to a defective pentose phosphate pathway (Figure 2), but methylene blue administration can induce haemolytic anaemia in these patients.^63^

A range of alternative treatments should be considered in methaemoglobinaemia cases when methylene blue is contraindicated, unavailable or ineffective. High dose vitamin C has also been shown to be effective at reducing MetHb levels.^64^, ^65^ Therapeutic whole blood exchange has been found to be a safe and effective treatment for adult methaemoglobinaemia—particularly in refractory cases or when methylene blue is contraindicated.^61^ Hyperbaric oxygen has also been reported to be effective in refractory cases.^66^ The evidence supporting the use of these alternative treatments remains largely observational—further research into their use as second‐line treatments is warranted.

As discussed above, over half the reported cases of methaemoglobinaemia between 1980 and 2020 were due to aniline group‐containing agents, namely dapsone and cocaine‐derived anaesthetics.^37^ Research into the metabolism of these agents is ongoing: they are likely metabolised to hydroxylamines.^39^ Hydroxylamine metabolites can be recycled after oxidising Hb, so continue to act after initial methylene blue treatment. Patients may have a temporary reduction in MetHb levels, only to ‘relapse’ back to toxic levels due to the continuous cycle of hydroxylamines.^67^ This mechanism likely explains the higher frequency of repeat methylene blue dosing observed in dapsone‐associated cases.^38^ Charcoal has been recommended in these cases for decontamination.^68^ P450 inhibitors can block the formation of hydroxylamines from dapsone in vitro.^69^ Due to the frequency of cases caused by aniline group‐containing compounds (Figure 3), further research on the effectiveness of P450 inhibitors at preventing hydroxylamine and MetHb formation would be useful.^37^ Cimetidine prevents the formation of dapsone‐hydroxylamine in vivo and shows clinical potential at preventing MetHb formation in patients undergoing long‐term dapsone therapy.^70^

Recent data have highlighted variability in healthcare system preparedness for treating patients with methaemoglobinaemia: significant disparities in the availability of real‐time co‐oximetry and methylene blue are reported across institutions, particularly between urban and rural settings.^71^ These findings suggest that delays in diagnosis and treatment may occur in some settings, despite established management strategies.

## CONCLUSIONS

Methaemoglobinaemia is a rare but clinically important disorder with diverse inherited and acquired causes. It should be considered in patients presenting with cyanosis or hypoxaemia that is disproportionate to the measured PaO_2_ or unresponsive to oxygen therapy. MetHb concentration influences clinical presentation and should guide management.

Recent evidence supports the efficacy and safety of intravenous methylene blue as the cornerstone of treatment for symptomatic methaemoglobinaemia, with high‐dose vitamin C and exchange transfusion serving as important adjunctive or second‐line therapies in selected cases. Emerging data suggest that the epidemiology of acquired methaemoglobinaemia is evolving, with an increasing contribution from recreational and non‐prescribed exposures in contemporary practice.

Despite well‐established treatment strategies, challenges remain in the timely recognition and management of methaemoglobinaemia, particularly in settings with limited access to co‐oximetry or anti‐dotal therapy. Future research priorities should include the development of prospective registries to better characterise disease patterns and outcomes, improved availability of diagnostic testing, and higher‐quality evidence to guide second‐line and adjunctive therapies. Further research into preventative strategies, including modulation of oxidant metabolism, may offer opportunities to reduce the burden of disease in at‐risk populations.

## AUTHOR CONTRIBUTIONS

**David C. Rees:** Conceptualization; writing – review and editing. **Alexander J. Twine:** Conceptualization; writing – original draft; writing – review and editing.

## FUNDING INFORMATION

The authors have nothing to report.

## CONFLICT OF INTEREST STATEMENT

The authors have no conflicts of interest to disclose.

## ETHICS STATEMENT

Not applicable.

## PATIENT CONSENT STATEMENT

Not applicable.

## PERMISSION TO REPRODUCE MATERIAL FROM OTHER SOURCES

Not applicable.

## CLINICAL TRIAL REGISTRATION

Not applicable.

## Data Availability

Not applicable.
